# The effect of cochlear implantation on autonomy, participation and work in postlingually deafened adults: a scoping review

**DOI:** 10.1007/s00405-020-06490-x

**Published:** 2020-11-27

**Authors:** Hugo G. B. Nijmeijer, Noud M. Keijsers, Wendy J. Huinck, Emmanuel A. M. Mylanus

**Affiliations:** 1 Department of Otorhinolaryngology and Head and Neck Surgery, Radboud university medical center, P.O. Box 9101, Nijmegen, 6500 HB The Netherlands; 2 Donders Institute for Brain, Cognition and Behavior, Radboud university medical center, P.O. Box 9101, Nijmegen, 6500 HB The Netherlands

**Keywords:** Cochlear implant, Hearing loss, Work, Autonomy, Participation

## Abstract

**Purpose:**

This scoping review examines the available evidence on the effect of unilateral cochlear implantation (CI) in adults with postlingual bilateral hearing loss on societal-related outcomes in terms of work, autonomy and participation.

**Methods:**

Five databases were searched (Pubmed, Web of Science, Embase, PsycINFO and Cochrane Library). Publications were screened in three steps on inclusion criteria. Of the 4230 screened publications, 110 met the inclusion criteria and were assessed for data extraction regarding outcomes “work”, “autonomy”, “participation”. Study characteristics and key findings are presented and narratively described.

**Results:**

Twenty-seven publications were included and categorized into retrospective (*n* = 3), cross-sectional (*n* = 18) or prospective (*n* = 6) study designs. Measurement or identification of number of outcomes (no) were related to work (no = 20), participation (no = 9) and autonomy or independency (no = 10). Most studies indicated benefits of CI on these outcomes. However, some studies did not or indicated additional barriers for benefits. Eleven publications primarily aimed to study one or more of our primary outcomes.

**Conclusion:**

In this literature search, scientific databases are reviewed. The results indicate that there is a relatively small body of evidence regarding the effect of CI on the outcomes “work”, “autonomy” and “participation”. Even though there are some limitations of the current study including some overlap in outcome definitions, most included studies indicate a beneficial effect of CI on work, autonomy and participation. The lack of consensus in definitions and the small body of evidence indicates a need for additional prospective studies investigating the societal outcomes of CI in postlingually deafened adults.

**Electronic supplementary material:**

The online version of this article (10.1007/s00405-020-06490-x) contains supplementary material, which is available to authorized users.

## Introduction

Hearing loss in adults gives rise to limitation in communication, with evident negative consequences in daily life [1]. In 2017, the World Health Organization (WHO) presented a report on the prevention of hearing loss that states that adults with unaddressed hearing loss have higher unemployment rates than the rest of the population. Daily limitations in hearing and understanding speech lead to fatigue and more recovery time from work [2, 3]. Furthermore, older adults with hearing loss face significant physical and social challenges, their hearing disability contributes to social isolation and loss of autonomy with associated anxiety, depression, cognitive deficits and dementia [4, 5].

There are multiple causes of hearing loss and—depending on the type and cause of hearing loss—it is often progressive in nature [6]. The WHO defines hearing loss by ISO 4PTAdB hearing loss as follows: slight/mild = 26–40 dB; moderate = 41–60 dB; severe = 61–81 dB; profound > 81 dB [7]. At the levels of severe and profound hearing loss, the use of hearing aids might no longer result in hearing benefits. Following natural progression, these patients would often rely on sign language and lipreading.

However, since a couple of decades, rehabilitation of severe to profoundly deaf individuals is possible with cochlear implants (CIs). CI has demonstrated to improve audition, speech perception and health-related quality of life (HRQoL). Unilateral cochlear implantation has proven to be a cost-effective intervention for rehabilitation of patients with profound bilateral sensorineural hearing loss [8]. Till now, alternatives to CI for rehabilitation of these profound cases of hearing loss is limited to educational programs for the deaf [6].

CI efficacy and effectiveness have been demonstrated in clinical studies, often conducted in controlled laboratory setups (anechoic soundproof booths). This has often been studied in clinical settings using psycho-acoustic measures like speech perception in noise and sound localization tests. In addition, changes in HRQoL have frequently been evaluated using generic [for example the Health Utility Index Mark lll (HUI3)] and disease-specific [for example Nijmegen Cochlear Implant Questionnaire (NCIQ)] health-related quality of life questionnaires.

However, besides health-related quality of life, audition and communication, there are other interrelated, important aspects for an individual who participates in a complex society. The experienced benefit of the patient in society may differ from their clinical audiologic improvement. Measuring these societal outcomes reveals information about the value of CI for the individual and society as a whole. Proper understanding of the societal benefit of CI may help justify its budgetary burden, which is bound to increase as a result of demographic changes. The Dutch National Institute for Public Health and the Environment (RIVM) projects a scenario of 48% increase in absolute number of patients with hearing disorders between 2015 and 2040 [9].

In scientific and grey literature, these societal-related benefits of CI have often been addressed theoretically, but have not always been studied in a systematic manner. We are interested in the current body of evidence regarding societal-related outcomes which we further specified as work; autonomy; participation; quality of life (QoL), cognition and communication. Conducting a scoping review is suitable to provide an overview of the existing evidence and report on the types of evidence that investigates the effect of CI on these societal-related outcomes [10].

The aim of this scoping review is to examine the extent of available research regarding the effect of unilateral cochlear implantation in adults with postlingual bilateral hearing loss on societal-related outcomes in terms of work, autonomy, and participation. This is of importance to guide future research and/or inform policy makers about cochlear implantation and for the counseling of individual patients.

## Methods

This scoping review was conducted based on the methodological framework described in the Joanna Briggs Institute Reviewers manual [10]. Reporting was based on the Preferred Reporting Items for Systematic reviews and Meta-Analyses extension for Scoping Reviews (PRISMA-ScR) checklist [11].

### Eligibility criteria

In the search strategy of this study, the following search criteria were used.

#### Population

Adults (≥ 18 years of age) with severe and profound hearing loss according to the WHO classification (severe: 61–80 dB HL, profound: > 81 dB HL) or complete deafness were included. We excluded studies in which participants were only patients with specific underlying disorders or co-morbidities, such as blindness or Parkinson’s disease. Publications were excluded if they exclusively investigated animals, children, prelingually deaf adults, single-sided deafness (SSD) patients or bilaterally implanted patients.

#### Intervention and comparator

All publications investigating the effect of cochlear implants, bimodal and electric acoustic stimulation (EAS) fitting were included. Studies without a comparison between patients with and patients without unilateral CI or exclusively studying bilateral implantation were excluded. Studies that investigated interventions post CI surgery to improve CI outcomes were excluded. In addition, studies exclusively investigating surgical procedures, imaging, CI device specifications, fitting procedures and (patho)physiology were excluded.

#### Outcomes

*Autonomy* is defined as the ability of living the way you want without being dependent on other people, independence. *Participation* is defined as the ability to participate or participating in (social) events and activities that an individual wants to attend. *Work* is described as being employed and how this employment is being experienced, as well as being engaged in activities that are perceived either by the CI recipient or by co-workers as work. In the present review, the outcomes are considered as relevant if the authors of the particular study defined and measured this outcome as such. In case of qualitative studies, the results should be thematically coded in terms of these outcomes. In addition, we also searched for outcomes related to cognition, QoL and communication because these outcomes were considered related to our primary outcomes of interest. *Cognition* is not further specified; however we included memory separately in the search strategy. (*Health-related*) *Quality of life* is also included. *Communication* is defined as observed or self-perceived, subjective changes in communication as a result of CI. Studies that did not measure or describe one of the above outcomes were excluded. Clinical audiometric tests were excluded as those may not always correlate perfectly with perceived benefits by the patient [12].

All publications investigating the effects of unilateral CI in severe to profound postlingually deaf adults on participation, work, autonomy, QoL, communication or cognition were assessed. Also publications that focused on different primary outcome measures, but did present relevant data on the outcomes work, autonomy and participation, were assessed. Relevant reviews which did not generate new data, for instance through a meta-analysis, as well as relevant protocols of ongoing studies were kept separately. We did not put constraints on publication year. Studies written in any language other than English or Dutch were excluded.

### Information sources and search

Five scientific databases (Pubmed, Web of Science, Embase, PsycINFO and Cochrane Library) were searched. A search strategy was developed and finalized by consulting a librarian from the Radboud University’s medical library. The used search strategies are shown in Online Appendix 1. Duplicates were removed by using Endnote citation software. Included publications were categorized based on the investigated outcome.

### Study selection process

Literature screening was performed in three steps. Two screening phases were based on title and abstract and one comprised full text screening. All screening rounds were performed by two researchers (HN and NK). In the first screening round, the publications were divided over the two researchers, because of feasibility and the breadth of the initial search. This screening round was conducted with most caution, meaning that only publications that did not meet inclusion criteria were excluded. In the second screening round, all remaining publications were exported to the literature screening software Rayyan [13], and each article was independently screened by both researchers for inclusion based on title and abstract. Conflicts were discussed till consensus was reached. The third and final screening round was also performed in Rayyan. All remaining publications were screened on full text by the two main researchers independently. As before, conflicts were discussed and solved until consensus was reached. In addition, all included publications were labeled for summarizing characteristics and data extraction.

Since this review focusses on participation, work and autonomy, no data extraction was conducted on studies solely investigating QoL, communication and cognition. The most important recent systematic reviews regarding QoL, cognition and communication are briefly described in this review.

The first search was conducted on 4-6-2019. An update search on the outcome measures work, participation and autonomy was conducted on 15-4-2020. The retrieved publications of the updated search were independently screened in two phases, title/abstract screening and full text screening, by both researchers. When there was conflict regarding inclusion, the researchers discussed until consensus was reached.

### Data extraction process and synthesis

After screening, all included publications measuring or identifying participation, work or autonomy were used for data extraction. Before data extraction, a data extraction form was created. The form was tested on three publications by both researchers. The results of this test were then compared and discussed. Small adjustments were made to the data extraction form accordingly. Once all researchers agreed on the data extraction form, it was used for data extraction of all included studies. This was performed by dividing the studies over the two researchers. After completion, the researchers checked each other’s work and conflicts were discussed until consensus was reached. During data extraction, the researchers maintained the used data as close as possible to the official text of the article, to ensure that the original author’s nuance in claims remained intact. Because study design was not often stated explicitly, we categorized them according to retrospective, cross-sectional and prospective studies. The categories were defined according to when the study started, how often they measured outcomes and for what initial purpose. Retrospective: one or multiple measurements before the start of the described study or the data was initially used for another purpose/research. Cross-sectional: one measurement point in time after start of study for current study goals. Prospective: multiple prospective measurements at multiple time points after the start of the study and data is used for current study goals. The used data extraction form is shown in Online Appendix 2. Results of this data extraction are presented in a summary table and described narratively.

## Results

**Fig. 1:**
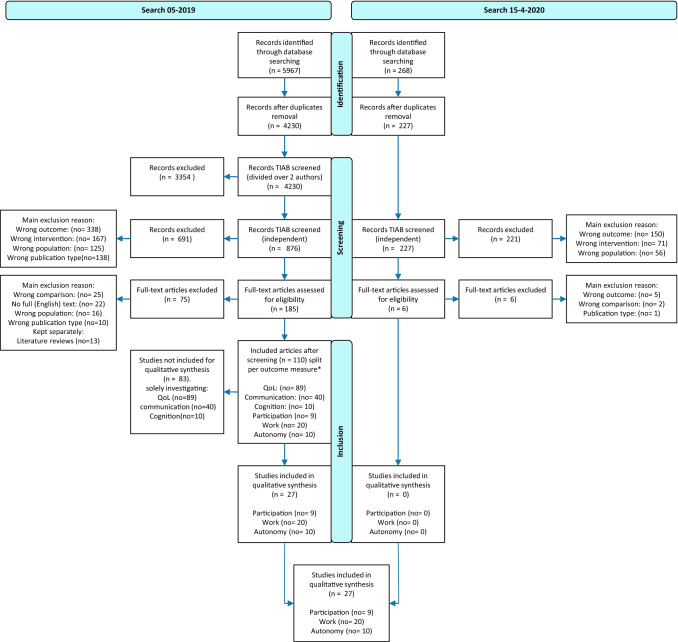
Flowchart of the search strategy. Most frequent exclusion criteria are mentioned. The number of studies is indicated by ‘*n*’. Number of outcome measures investigated in the studies and if multiple exclusion reasons are applicable are indicated by ‘no’. The total number of ‘no’ can be higher than ‘*n*’

The search strategy used in Pubmed is shown in Online Appendix 1. This search method is adapted for use in Web of Science, Embase, PsycINFO and Cochrane Library. Initially, a total of 4230 unique publications were found. Due to the broad search strategy, this included a lot of studies unfit for the present study. After the first screening round based on title and abstract, 876 publications remained. Following the second screening round, 691 additional publications were excluded. The main reasons for exclusion in this phase were based on outcome (no = 338), intervention (no = 167), wrong publication type (no = 138) or population (no = 125). Finally, the remaining 185 publications were included in the full text screening, after which 75 publications were excluded. In the full text screening, the three main exclusion reasons were a wrong comparison (no = 25), no full (English) text being available (no = 22), literature reviews (no = 13), wrong publication type (no = 10) or incorrect population (no = 16). The update search on 15-4-2020 yielded 227 publications after removing duplicates. After title and abstract screening, six publications remained for full text screening. None of the publications eventually met our inclusion criteria. The flowchart is shown in Fig. 1. Some studies investigated multiple outcomes, hence the total number of included studies is lower than the sum of total number of included studies per outcome measure. For clarification, number of studies will be indicated with “*n*”, whereas number of outcome measures or exclusion reasons will be indicated with “no”.

Twenty-seven publications reporting on participation, work or autonomy were included for the data extraction in which the outcomes of interest were measured or identified for a total of 39 times. Of these 39 outcomes, 9 included measurements about participation, 10 about autonomy and 20 about work. Unlike a systematic review, studies of various study designs can be included in a scoping review. In terms of study design, no randomized controlled clinical trials were found with the used search strategy and selection criteria. Three of the included studies were retrospective studies, 18 were cross-sectional studies, and 6 were prospective studies. The type of data investigated was categorized as qualitative in 9 studies, quantitative in 17 studies and 1 study used a mixed method approach. As there were no restrictions regarding publication date, we included 5 publications with a publication date before the year 2000, 10 studies between 2000 and 2010 and 12 studies after 2010. Although no restrictions were used for continent or country of the studies, all included studies were published in Europe, North America or Australia. The study characteristics are presented in Table 1.

**Table 1:** Study characteristics: number of included publications per societal outcome measure, type of study, year of publication and continent

	Number of each measured outcome (total no = 39)
Included outcome measure	
Participation	9
Autonomy	10
Work	20
	Number of included studies (*n* = 27)
Type of study	
Observational	
Retrospective	3
Cross-sectional	18
Prospective	6
Interventional (RCT)	0
Type of data	
Quantitative	17
Qualitative	9
Mixed methods	1
Year of publication	
Before 2000	5
2000–2010	10
2010–2020	12
Continent	
Europe	16
North America	6
Australia	5

The key findings of the included studies are shown in Table 2. The entire completed data extraction form is shown in Online Appendix 2.

**Table 2:** Summary of data extraction. Key methodology and findings are shown per included study

Author and year (references)	Outcome(s) label	Number of participants	Methodology^d^	Measurement tool	Key findings
Chapman 2017 [15]	Work, participation^a^	801 (254 with CI, 547 without CI)	Cross-sectional analysis^c^	Self-made questionnaire	CI group reported higher levels of feeling limited than those without CI. Without CI more likely to participate in deaf culture activities. With CI more likely to socialize with hearing people. Higher reported social participation in mainstream organizational activities, in the group without CI than with CI. Regarding work, participants without a CI were significantly more likely than those with CI to report cultural differences as a challenge
Clinkard 2015 [33]	Work^a^	65 CI	Cross-sectional study^b^	Self-made questionnaire	31% reported increase in income > 20.000 after CI. Employment was increased from 40 to 49 (out of 65). An increase in employment of 22% may also reduce the utilization of social and disability assistance by these patients
Czerniejewska-Wolska 2015 [34]	Work	20 CI	Retrospective qualitative study^c^	Not specified	Average age at implantation was 67.8 years. 14 individuals are retired, one is a half-time worker and 2 subjects are full-time workers who are willing to continue working. Three subjects were neither retired nor working. As self-assessed, hearing impairment sometimes or always affected their work before surgery. After 1 year of using the CI, 2 patients indicated improvement in hearing due to the CI that led to better professional performance. One patient reported no change in work performance
Fazel 2007 [35]	Work^a^	65 CI	Cross-sectional study^b^	Unspecified standardized questionnaire	The cochlear implantation group showed a positive impact on employment status and prospects. The likelihood of being employed increased post-implantation. CI also contributes to a higher job satisfaction and improves the recipient’s perception of his/her employment opportunity
Härkönnen 2017 [36]	Work^a^	8 hybrid CI	Cross-sectional study^b^	Unvalidated questionnaire measuring working performance	Hybrid CIs and conventional CIs had a positive impact on their well-being and working performance. Patients managed better at work and the implantation had a positive influence on their career planning. Easier communication with co-workers. Patients were more active in working environment and more alert after the working day. In all comparisons groups, working performance increased. Our results may reflect individual experience and performance, and outcomes may not be directly derived from the CI treatment option
Hawthorn 2004 [23]	Participation^a^	34 HI	Prospective study	Hearing Participation Scale (HPS)	Large positive effect on social participation over a 6 month follow-up after CI
Hogan 1997 [16]	Work, participation	18 CI users were interviewed. 129 CI users, 78 severe to profound HI and 33 deafened adults filled in the questionnaire	Cross-sectional mixed method study	Semi-structured interviews and a brief 14 point, socioeconomic status questionnaire, supplemented with socio-demographic items. The Code–Muller protocol-Revised (revised for this study)	Cochlear implantation can result in substantive, interpersonal and social gains for deafened people. However, employment outcomes did not improve for respondents following implantation. A barrier for improved CI employment outcomes were identified in poor or lack of referral between CI program and government employment or training services
Hogan 2001 [22]	Participation^a^ autonomy	202 (148 CI, 54 no-CI) and 136 partners (105 from implantees, and 31 from non-implantees.)	Cross-sectional study	Participation Scale (PS) and the Assessment of Quality of Life (AQoL) subscale independent living	Both unadjusted and adjusted score results suggest that cochlear implants have a large impact on recipients’ social participation. No differences were found on the subscale independent living
Hogan 2002 [37]	Work^a^	12 CI	Cross-sectional qualitative study^b^	Focus group with semi-structured interview	Cochlear implantation has resulted in significant benefits to deafened adults in the workplace. Respondents indicated that, as a result of implantation, they are more confident and independent in the workplace. They report greater job satisfaction and prospects for their future. In addition there are a range of issues that facilitate access to and retention of employment. To achieve such outcomes, there should be combination between technology and the social processes, and the device itself does not create better jobs
Huarte 2016 [20]	Work^a^	60 CI	Cross-sectional study ^b^	Retrospective Working Life Satisfaction Questionnaire (rWLSCI) for patients implanted with a CI	Fifty of the 60 respondents were actively employed. Twelve of them had found a job after implantation, of which 5 indicated this was due to implantation. CIs are very useful in enhancing working life and social skills because they encourage implanted patients' ability to communicate
Kos 2006 [18]	Work^a^	60 CI	Cross-sectional study^b^	An unspecified questionnaire focusing on their professional activities	Implantees may retain and develop their professional skills. Implantees may also progress in the development of new nonprofessional activities. However, CI may still be perceived as insufficient to enable satisfactory hearing to facilitate further learning or career development—not by the implantees, but by the potential employers. Furthermore, it is not valid to assume that CI recipients who maintained their job would have stopped if they did not receive an implant
Lawchowska 2013 [38]	Autonomy	31 CI	Retrospective study	Interview-style questions	Elderly patients gained more independence after implantation as a result of improved communication, offering them a chance to become more active in everyday life
Looi 2011 [39]	Work	94 CI and 70 CI candidates on waiting list	Cross-sectional study	Questionnaire, partly consisting of Nijmegen Cochlear Implant Questionnaire (NCIQ) and Cochlear Implant Satisfaction Questionnaire (CISQ)	The CI was associated with increased participation in employment. Question 6 of the questionnaire: In the waiting list group 51% felt that their hearing impairment caused difficulties in their work and studies. This was similar for only 5% of the CI group. The CI was also associated with increased job satisfaction and broadened employment opportunities
Maki-Torkko 2014 [26]	Work, autonomy, participation	101 CI and 87 significant others	Cross-sectional qualitative study^b^	Self-made open-ended questionnaire for patient and significant other covering areas such as positive and negative factors related to the implantation	The CI increases well-being and satisfaction in life for both CI users and their significant others, especially regarding enhanced autonomy, normality and everyday social life. The study did not aim at generating a theory, and cannot be generalized to other contexts. Some CI users reported to perform work tasks that were impossible before implantation (information desk) or able to perform tasks just like anyone else
Mo 2004 [40]	Work	82 CI. 59 HA, and 35 no CI users (A: *n* = 19 did not meet criteria, B: *n* = 16 rejected for CI due to good hearing levels)	Cross-sectional study	Subscale of the Patient Quality of Life Form	In the subscale categories concerning relations to close individuals, work, and hobbies, there were no significant differences
Mo 2005 [14]	Work	27 postlingually deafened CI candidates. Before surgery, 12 and 15 months after surgery	Prospective study	Subscale of the Patient Quality of Life Form	In the subscale categories “work” and “hobbies,” the differences were not statistically significant; 18 and 13 [participants] improved, 5 were unchanged in both categories, and 4 and 9 became worse after surgery, respectively. CI recipients significantly improved on the ability to communicate, were less isolated, less feeling of being a burden and better relations with close friends
Monteiro 2012 [19]	Work^a^	381 CI	Retrospective study	Self-made questionnaire	Unilateral CI appears to result in benefits from an income and economic perspective. This benefit helps offset initial societal costs of implantation, but may also benefit society through an increased taxation pool
Neria 2011 [41]	Autonomy	5 CI adults. (Implanted at childhood or were teenagers at time of implant)	Cross-sectional Qualitative study	Multiple in-depth open-ended interviews. Using phenomenology as a theoretical framework	CI is likely to increase autonomy in deaf emerging adults
Rembar 2009 [21]	Work, participation, autonomy	74 CI	Cross-sectional qualitative study	Self-made open-ended questionnaire regarding shortcomings and benefits of CI	The subjects’ employment situation had generally improved, as they either performed better at the job they had or got a better job. Also positive effect on education, as well as on everyday activities. Social participation increased. However, the implant presented a limitation to certain physical activities. Some indicated an increased feeling of independence. On the negative side, some were met with too high expectations from others
Rihkanen 1990 [42]	Work	28 postlingually deaf adults. 10 received a CI	Prospective study	Unspecified questionnaire, including multiple choice questions	During follow-up, 2 participants in the CI group retired. One CI user had returned to work and another CI user had taken a part-time job. They both felt that the implant made the re-employment possible. Limitations were related to small number of subjects, lack of random selection and modern surgical intervention that might have influenced subjective attitude
Ross 2007 [17]	Work	6 CI (1 participant had prelingual hearing loss)	Cross-sectional qualitative study^b^	Semi-structured interviews with patients and their partners. Participants had influence on the agenda of the interview	After CI, social interactions gradually increased, both with and without the partner. Improvements were noticed in self-confidence. Not all working CI recipients experienced improvements in the hearing-related issues at work, largely due to the unrealistic expectations from their employers, who expected once implanted normal hearing would be restored
Saxon 2001 [43]	Work^a^	13 CI (9 employed, 4 retired or self-employed) and 9 employers	Cross-sectional design^b^	Abbreviated Profile of Hearing Aid Benefit (APHAB) questionnaire modified for CI and work	Results indicate that CI has a positive impact on job functioning, particularly through improved communication and ability to identify warning signals. In terms of recommendations: CI clients and work supervisors should be counseled on the potential benefits and limitations of cochlear implants in the workplace
Sonnet 2017 [25]	Autonomy	16 HI Before surgery. 6 and 12 months after surgery	Prospective study	Autonomy measured by four criteria of the Instrumental Activities of Daily Living (I-ADL) questionnaire (telephone use, transportation, medication and domestic finances)	This study shows that CI in elderly patients can improve their speech understanding and their autonomy, without deterioration of their cognitive functions. I-ADL scores remained stable over 6 months. Scores significantly improved after 12 months
Tyler 1990 [27]	Work, autonomy, participation	53 better CI users	Cross qualitative sectional study^b^	Open-ended self-made questionnaire	No clear narrative conclusion regarding these outcomes. The study investigated the advantages and disadvantages. Fourteen patients reported an increase in participation in social activities, 10 reported improvements at work and 9 reported an increase in independence. Five patients mentioned an acceptance by others/no longer isolated. This study only included successful CI users, meaning that results favor benefit and minimize problems. However, the advantages and disadvantages mentioned might still be applicable for a more representative sample
Völter 2018 [24]	Participation, autonomy	60 pre-implantation, 33 at 6 months follow-up, 20 at 12 months follow-up	Prospective study	Autonomy and participation subscales of the WHOQOL-OLD	No clear narrative conclusion regarding these outcomes. Comparing 6 months post-op to pre-op they observed statistical differences on the autonomy scale, and no statistical significance on the social participation scale. Comparing 12–6 months post-op there were no further improvements
Wexler 1982 [44]	Work, participation, autonomy	22 CI users and 13 index relatives	Cross-sectional study^b^	Self-made questionnaire based on an earlier interview study with CI users	For profoundly deafened, the implant increases the sense of security, diminishes dependence on others, reduces the frequency and intensity of emotional distress, promotes communication, particularly through more efficient speechreading, and there is a significant decrease in dread when social situations are encountered. These outcomes are confirmed by index relatives. Participation in group activities is still difficult, but less threatening. Although no significant differences observed, many patients indicated that both interest and comfort in employment improved compared to pre-implantation
Zhao 1997 [45]	Autonomy	26 HI (of these 13 were fitted with a CI)	Prospective qualitative study	Open-ended hearing questionnaire as developed by Barcham et al. The benefits/problems questionnaire for patients with a CI	CI users reported significantly more benefit than in literature reporting HA or BAHA users, but equal shortcomings of the device. Of the CI users, 38% reported feeling less isolated from society and 15% reported having become more independent since the implantation

^a^Indicates that this outcome was one of the primary study outcomes

^b^Indicates cross-sectional studies in which patients had to recall experiences or states to the time before CI

^c^Part of a nationwide survey study or a larger observational database project

^d^If methodology was not clearly described, it was deduced and categorized as retrospective, cross-sectional or prospective

### Work

We found 20 studies in which the effect of CI on work was measured or identified. Of these studies, there were 16 cross-sectional studies (10 quantitative data, 5 qualitative data and 1 mixed methods), 2 prospective (quantitative) studies and 2 retrospective (1 quantitative and 1 qualitative) studies. Only eight studies investigated work or employment as a primary objective. The other 11 studies were either for developing a methodological tool, a secondary objective or they reported on a subscale or item of a questionnaire. Overall, most studies indicate some improvement in working performance, employment status or income after cochlear implantation. However, some studies did not observe a clear difference. The prospective quantitative study by Mo et al. assessed 27 postlingually deafened CI candidates. In this study, data were obtained pre surgery, and 12 and 15 months after surgery [14]. They showed an improvement in HRQoL on the Patient Quality of Life Form (PQLF) [14]. However, regarding the PQLF subscale “work”, there were no significant differences reported. Even though 18 participants improved, 5 were unchanged and 4 scored worse after surgery on the ‘work’ subscale [14]. Chapman et al. 2017 conducted a cross-sectional study, which was part of the Danish national survey, and compared CI recipients (*n* = 254) with hearing-impaired individuals without a CI (*n* = 574) [15]. Regarding the domain “challenges at work” with hearing people, they showed that people without a CI were statistical significant more likely to report cultural differences as a challenge. In the other challenges at work domains, there were no differences between the CI and no CI group on communication cooperation, isolation, social issues, other and no problems. Hogan 1997 applied a mixed method design in which they interviewed 18 CI recipients and provided a 14-item questionnaire regarding socioeconomic status to 129 CI users, 78 patients with severe hearing loss and 33 deafened adults [16]. Implantees had a CI for an average of 7 years. According to this study, 91% of the implantees reported ‘no change’ pre- and post-implant. In addition, they identified that the group of implantees reported no higher incomes compared to their peers without CI [16]. However, the implant rehabilitation program did not include employment support. For the CI recipients who remained or retained at their job experienced an improvement in the way they were treated [16]. The study of Ross et al. 2007, in which they interviewed six CI recipients, identified that not all working CI recipients experienced an improvement at work, mainly due to their employers, who expected that after implantation the patient’s normal hearing would be restored [17]. Finally, the quantitative cross-sectional study of Kos et al. 2007 concluded that although CI users may retain and develop their professional skills, potential employers may still perceive CI as insufficient to enable satisfactory hearing to facilitate further learning or career development [18].

Monteiro et al. 2012 conducted a retrospective study in which they used data from originally included 637 hearing-impaired patients, 45.3% of which reported to be employed at the time of diagnosis of hearing loss. Of the 301 employed patients at the time of hearing loss diagnosis (who provided sufficient data), 36.7% reported to have suffered negative impact on employment as a result of their hearing loss (20% lost job; 9.5% early retirement; 7.2% long-term disability). At the time of initial assessment for CI, 269 subjects reported to be employed; this resembles a 5% decrease in employment compared to the employment rates at the time of hearing-impaired diagnosis [19]. Only 381 patients provided sufficient data in the follow-up period post-implantation for analysis. After implantation, 51.1% of the patients reported to be employed, which was an increase of 10.8% compared to the initial assessment. 34.2% of the patients reported a change in their employment after implantation, of which 77.8% reported a positive change in employment status, compared to 22.2% who either suffered a negative impact on employment or elected to retire shortly following implantation. Of the subjects who sustained a positive change in employment status following cochlear implantation, 83.8% felt that this was attributable to being implanted [19]. Huarte et al. 2017 conducted a cross-sectional study in which 60 CI recipients with a mean age of 48 years were included to retrospectively asses to what extent CI affected their working lives [20]. 83.3% were actively employed at the time of completing the questionnaire. 12 participants (20%) indicated to have found employment after implantation, and 5 of those 12 attributed this to receiving the implant. In this study, 41.2% of the respondents felt a decrease in discrimination at work after cochlear implantation. This might be in line with the findings of scores of cultural challenges presented by Chapman 2017 [15]. Finally, the qualitative cross-sectional study of Rembar et al., who used a self-made open-ended questionnaire (consisting of four questions) regarding shortcomings and benefits of CI, observed the subjects’ employment situation had generally improved, as they either performed better at the job they had or got a better job [21]. Similar to the study of Ross et al., some patients reported they were met with too high expectations from others [17].

### Participation

In total, nine studies investigated or identified (social) participation as one of their outcomes. Two of these were prospective quantitative studies. The other seven studies were cross-sectional studies, three used quantitative methods, three used qualitative methods and one used a mixed methods approach. Only three studies explicitly aimed to investigate participation as part of the primary objective, the remaining six were either identified as participation during the research or were used to develop a research tool. Most of the identified studies suggest a positive effect of cochlear implantation on (social) participation. However, the national-based cross-sectional study of Chapman et al. showed surprising results regarding participation [15]. When analyzing all participants, they observed that CI recipients (*n* = 254) reported to be more limited by their hearing loss and reported to participate statistical significantly less in mainstream organizational activities compared to participants without implantation (*n* = 547). When divided over age groups, similar results were found regarding the participation in mainstream organizations scores in the younger than 26 years group, but there were no significant differences found in the older than 25 years group [15]. However, in the analysis of all participants and the group older than 25 years they reported that CI recipients were more likely to socialize with hearing friends compared to participants without a CI [15]. The cross-sectional study of Hogan et al. 2001 [22] is the second largest included study after Chapman et al. They included 148 CI recipients and 54 no-CI recipients with no statistical difference between time since onset of deafness between the groups. Hogan et al. used the Participation Scale (PS), which was derived from the Glasgow Health Status Inventory. They observed an overall significant increase of 29% on PS comparing implantee versus non-implantee [22]. According to the author, this suggests that CI has a large impact on a person’s social participation. In the prospective study of Hawthorn et al., they included 34 participants who completed the Hearing and Participation Scale (HPS) before surgery and with a follow-up of 3 and 6 months after surgery. There were significant improvements on the HPS score between baseline and 3 months follow-up and between 3 and 6 months follow-up [23]. The other prospective study of Völter et al. reported scores of the social participation subscale of the generic quality of life tool WHOQOL-OLD [24]. 60 participants provided data prior to implantation, 33 of these were reassessed 6 months after implantation and 20 participants were assessed after 12 months. Even though general QoL improved at 6 months after implantation, they did not find statistical significant improvement on the subscale social participation [24].

### Autonomy

Autonomy was measured or identified in ten studies. Three were classified as prospective studies, two quantitative and one qualitative. One study was a retrospective qualitative studies. The remaining six studies were cross-sectional, two quantitative and four qualitative, studies. None of these studies primarily aimed to gain specific insight into the outcomes autonomy or independence, meaning that all ten studies identified autonomy or independence during qualitative investigation, included it as part secondary outcome or it was part of subdomain score. Almost all studies that measured or identified perceptions relating to autonomy or independence suggest benefit after implantation. The quantitative prospective study of Sonnet 2017 was primarily interested in QoL and cognitive function in elderly patients (age: 65–80) [25]. They used the WHOQOL-OLD instrument, but did not report individual subscale scores. However, they also investigated autonomy by using the Instrumental Activities of Daily Living (I-ADL) questionnaire. Autonomy scores were assessed in 16 patients preoperatively and in 14 patients at 6 and 12 months after surgery. At 6 months after surgery, the autonomy scores were stable, with no statistical significant difference. The autonomy scores did improve at 12 months after surgery (mean 0.94 and *p* < 0.05) [25].

The other quantitative prospective study of Völter et al. 2018 did report the autonomy subscale of WHOQOL-OLD [24]. In this study, 60 patients were included pre-operatively, 33 completed assessment at 6 months follow-up and 20 completed the 12 months follow-up. General quality of life improved at 6 months after implantation, more specifically they also reported a statistical significant improvement WHOQOL-OLD autonomy subscale [24]. Maki et al. 2014 conducted a qualitative cross-sectional study in which they used self-made open-ended questionnaires for CI recipients (*n* = 101) and their significant others (family member or close friends, *n* = 87). This questionnaire covered areas such as pre-operative expectations and positive and negative factors related to the implantation [26]. They also investigated the perceived life changes and impact of the CI user’s significant other. They identified three subcategories: alienation–normality, fear–autonomy, living social life. These were considered to be interrelated and contributing to well-being and life satisfaction [26]. Both the CI users and significant others reported gain in autonomy, as the significant other no longer had to be a social bridge for the patient, reducing stress. CI users reported they could now handle social situations by themselves. The cross-sectional study of Tyler et al., investigated 53 “high performing” CI recipients. They also used a self-made open-ended questionnaire and showed that 9 of 53 patients reported an increase in independence after implantation [27].

### Communication, QoL and cognition

A total of 83 publications investigating QoL, cognition or communication, were included in the literature search; however most were not further analyzed for data extraction because they did not report on outcomes work, participation or autonomy. In these publications, there were some systematic reviews, with overlapping search results with our scoping review, especially regarding QoL and cognition. No systematic reviews were identified regarding the effect of CI on self-perceived communication by the patient. Although the systematic review of Gaylor et al., investigated communication-related outcome, but they maintained a different definition compared to the current review. They defined communication-related outcomes as the evaluation of speech perception (with either open-set sentence or word tests) [28]. The systematic review did conclude that unilateral CI is an effective method for improving speech perception and HRQoL [28].

McRackan et al. conducted two systematic reviews including meta-analysis, both in 2018 [29, 30]. The first review investigated hearing loss or CI-specific quality of life measures and the correlation with speech recognition ability [29]. In the current scoping review, we identified 11 of the 14 studies that were also included by McRackan et al. The difference of three studies is most likely due to differences in inclusion criteria. McRackan et al., concluded that their meta-analysis showed a large positive effect of cochlear implantation on QoL when using CI-specific patient-reported outcome measures (PROMs). In addition, the (pooled) correlation between speech recognition scores and hearing—or CI—specific QoL measures were considered negligible.

The other systematic review and meta-analysis by McRackan et al., investigated generic health-related quality of life measures [30]. In this systematic review, 22 publications met criteria for the meta-analysis. However, due to incomplete reporting regarding the statistics, only seven studies were used for meta-analysis. All these seven studies were identified, but not analyzed, in our current scoping review. The authors concluded that the meta-analysis showed a medium positive effect of CI on health-related quality of life. Similar to the earlier mentioned hearing-specific QoL measures, the correlation between speech recognition and generic QoL measures was considered negligible. In both the meta-analyses, generic and hearing/CI-specific QoL, a total of 16 unique studies were analyzed. In our scoping review, we identified a total of 91 studies regarding QoL. This discrepancy is probably caused by the different goals of the review and maintained inclusion and exclusion criteria.

Furthermore, Crowson et al., conducted a narrative review regarding the evidence of HRQoL and cost-effectiveness of unilateral and bilateral CI in children and adults. They provide a clear overview of design, used QoL measurement instruments and findings of some QoL studies also identified in our scoping review [31].

Claes et al. conducted a systematic review regarding cognitive outcomes [32], mainly focusing on older adults (aged 50 +) with bilateral hearing loss. Change in performance on cognitive tests was the primary outcome of interest. Studies using questionnaires were excluded and the included designs had to be longitudinal consisting of at least one pre- and one post-implantation measurement. Six studies met these criteria. All these studies were also identified in our literature search regarding cognition (*n* = 10). Five of these studies reported improvement in cognition after cochlear implantation [32]. However, in the discussion the authors stated that the studies had several risks of bias and were considered inconclusive. Claes et al., proposed the use of cognitive assessment tools that are adequately adapted to hearing-impaired individuals including a description of the modifications. To control for learning effects, they suggested using alternative forms. According to the authors, it was advised to perform more appropriate statistical analysis regarding the characteristics of the sample, descriptive values and outcomes.

## Discussion

In this scoping review, we aimed to explore the extent, measurements and types of studies that are available in scientific literature regarding unilateral cochlear implantation in adults with postlingual bilateral hearing loss on societal outcomes: work, autonomy, participation. In a total of 27 published studies, these outcomes were measured or reported 20, 10 and 9 times, respectively. Some studies included more than one of the above-mentioned outcome measures, hence the total number of measured outcomes (no) is larger than the total number of included studies (*n*). Insight is provided on the papers regarding autonomy, participation and work (*n* = 27). Most of the studies had a cross-sectional design (*n* = 18), some were retrospective studies (*n* = 3), while some were prospective (*n* = 6). The outcomes work, autonomy and participation were the primary objectives in 11 of the 27 studies. To prevent for missing papers that might have measured or identified work, autonomy and participation, the literature search was extended with the terms “quality of life”, “cognition” and “communication” which resulted in approximately six additional studies that would otherwise be excluded in title-abstract screening.

Most of the studies in this scoping review suggest a positive influence of CI on the outcomes work, autonomy and participation. However, some of the studies identified negative consequence of CI, mainly in terms of expectations by others. It was also indicated that it is important that CI recipients notify their communication partners as well as their employers and colleagues about the personal benefits and limitations that accompanies their CI use.

Based on this literature review, we would like to discuss several findings: (1) Generalizability and study year: It is known that qualitative study designs make generalizability difficult. Since some of the included publications in this review describe qualitative studies, conducted for exploratory purposes and to get more insight into the experiences of CI recipients, outcomes are less generalizable. Generalizability of study results to current practice is also impeded by the fact that some studies were rather old. Five of the included studies were over 20 years old. The participants in these studies had access to previous CI systems (including single channel CI) which may have provided incomparable results to the use of modern CI systems. A second issue with older studies is the assessment of a different study population in terms severity of hearing loss. This is the result of the fact that criteria for CI have broadened [46, 47]. Patients receiving a CI today frequently suffer from progressive hearing loss and have more residual hearing than patients who received a CI in the past. If a patient has no or very limited residual hearing, then there is more room for improvement which may make the older CIs look more effective than the newer ones. On the other hand, nowadays in general there is more residual hearing present pre-implantation and the time of auditive deprivation is shorter leading to better performance [46]. At this point, it is hard to determine whether these factors have resulted in an over- or underestimation of effects, but it is worth noting that the result from these older studies may differ from the more recent situation. (2) Many factors influence outcomes: When assessing or comparing studies regarding societal outcomes, it should be noted that more variables besides the CI device and surgery influence the results [35, 37]. Therefore, it is important to consider the entire CI procedure including aftercare and training services, mainly because there might be variation in this rehabilitation/aftercare program per country/center that could influence results on outcomes as work [16], autonomy, participation and probably QoL and daily living. (3) Methodology: Some limitations were reported regarding the methodology. A frequently reported limitation was a small sample size, followed by recall bias for retrospective- and cross-sectional studies. As shown in Table 1, no randomized controlled trials (RCT) were included. A possible explanation for this is that it is challenging to conduct an RCT with CI without ethical concerns [23]. There is no appropriate alternative treatment (that can serve as control group) for CI in patients with these levels of hearing loss [23, 40]. Comparison with another control group may not be valid because of variability in influential psychophysical and social characteristics, so they are not prognostically equivalent. Furthermore, many included studies used measurement tools that were either not specified or validated, which may create a risk for bias. Partially as a result of these limitations, studies commonly reported recommendation for larger-scale, prospectively longitudinal studies.

There are also some limitations in this review. Albeit the thorough and deliberate process of creating the search strategy, using a relatively large set of search terms, it might be possible that some synonymous terms were overlooked in our search. Publications that used slightly different terms were not found or identified during screening and might not have been included in this scoping review. However, if we missed any studies regarding these outcomes it is likely it does not drastically influence our findings and general direction of interpretation. Furthermore, the aim of scoping reviews is to give a broad, rather than in-depth, insight related to a particular research question. So, in this study we have been inclusive toward all study designs, including (retrospective, cross-sectional and prospective studies) various assessment tools and qualitative studies. In our review, we cannot determine if the included studies and their direction of results were the consequence of publication bias or non-reporting of negative results, which might be caused by the risk that significant results are more likely to be published or presented. In addition to including various types of studies, we included only studies that defined, either qualitatively coded or measured, specific outcomes. We identified the often implicit overlap and interrelation between definitions of our outcomes of interest (i.e., participation, QoL, autonomy) and specific measurement scales. It is therefore sometimes difficult to unravel the differences in definitions and what is actually being measured. The often lacking consensus around the definition of social participation and the consequence for analysis and policy was partially the rationale for Levasseur et al. to provide an inventory and content analysis of these varying definitions [48]. In our scoping review, this resulted in maintaining a strict terminology (defined it as participation autonomy or independence) and including studies in this review according to these terms. For example; the NCIQ is the disease-specific HRQoL questionnaire, which contains a domain regarding social interactions. This subscale was not included for data extraction in this literature study, for it was not specifically defined as participation, though questions or subscales regarding social interaction might be similar to those investigating participation. On the other hand, the study of Hawthorn and Hogan adjusted the Glasgow Health Status Inventory to create the shortened (Hearing and) Participation Scale (HPS and PS). These studies were included for it was explicitly stated to measure participation according to their definition. Furthermore, some general terms like quality of life consists of multiple domains, and an increase in quality of life does not entail that all the subdomains are improved, for example regarding the IPQLF which was included for further analysis because of the subscale ‘work’ [14, 40]. They did not show a statistical significant improvement in work, but the overall quality of life scale did show improvement in the CI group compared to one of the two no-CI groups. In line with this reasoning, when research is conducted, both generic and specific measures or subscales can be valuable to use for investigation. This mainly depends on the eventual purposes of the generated knowledge and how it will be used. For example, more generic normativebased instruments could be used in decision regarding resource allocation and comparison between different disease areas [49, 50]. Subscales and disease-specific tools might provide specific information in comparing relatively similar groups or for identifying specific targets for improvement. This should also preferably be driven by what is valuable to the patient from the concept of value-based health care.

In this scoping review, we provided an overview of the current body and level of evidence investigating the effect of cochlear implantation on the societal-related outcomes work, autonomy and participation. It can be concluded that there is a relatively small body of evidence that measured these societal outcomes, specifically there is a relatively small amount of prospective studies. On the other hand, a relatively large number of studies have been published (or recently reviewed) investigating QoL, cognition and communication.

Current literature review also underlines the interrelation between specific outcomes (i.e., autonomy, independence, participation) and subdomains of generic measures such as health-related quality of life measures. The included studies in this review investigated these outcomes separately. Making these outcomes explicit can be important for specifically guiding innovation and policy, to be able to provide better care by determining what is valuable for patients and eventually improving a patients’ quality of life. The value of these detailed explicit research outcomes depends on the decision-maker and end users of the research results.

In conclusion, based on the included studies in this scoping review, CI seems to have a beneficial effect on societal outcomes in terms of participation, autonomy and work. In times of increased scarcity of health-care budgets, this type of evidence is needed for policy and decision-makers. Today, the amount of robust scientific evidence regarding the effect of CI on the societal outcome measures is scarce. To overcome this omission, additional research on the societal outcomes of cochlear implantation in postlingually deafened adults is needed. Large prospective studies, using validated tools should overcome the most prevalent limitations (small sample size, non-validated tools and retrospective design) of the included studies in this review. These limitations greatly lowered the strength of the evidence of the included studies.

## Electronic supplementary material

Below is the link to the electronic supplementary material.

Appendix 1: Search strategy PubMed (XLSX 40 KB)

Appendix 2: Data extraction form (PDF 98 KB)

## Data Availability

Data are published or available upon request.
