# Supplementary material for: The effect of cochlear implantation on autonomy, participation and work in postlingually deafened adults: a scoping review
Source: Eur Arch Otorhinolaryngol. 2020 Nov 27;278(9):3135–54. doi: 10.1007/s00405-020-06490-x (PMC8328847; doi:10.1007/s00405-020-06490-x)
Supplement: Supplementary file 2 — Appendix 2: Data extraction form (PDF 98 KB) [file 405_2020_6490_MOESM2_ESM.pdf]

Appendix 1: search strategy

1A: search strategy pubmed; this strategy was translated to the other four databases.

Patient: NOT ((("Child"[Mesh] OR "Infant"[Mesh] OR "Adolescent"[Mesh]) NOT ("Adult"[Mesh])))
Intervention: ("Cochlear Implants"[Majr] OR Cochlear implantation[Majr] OR Cochlear implant*[ti] OR Cochlea implant*[ti] OR Cochlear Prosthe*[ti] OR Cochlea prosthe*[ti] OR Cochlear implant*[ot] OR Cochlea implant*[ot] OR Cochlear Prosthe*[ot] OR Cochlea prosthe*[ot])
Comparison: N/A
Outcomes:

- **Communication:** ("Communication"[Mesh] OR Communicat*[Ti] OR Communicat*[ot]) OR conversat*[Ti] OR Conversat*[ot])
- **Work**: (("Work"[Mesh] OR "Employment"[Mesh] OR "Occupational Health" [Mesh] OR work*[Ti] OR work*[ot] OR labour*[Ti] OR labour*[ot] OR labor*[ti] OR labor*[ot] OR employment[ti] OR employment[ot] OR unemployment[ti] OR unemployment[ot] OR Occupation*[ti] OR Occupation*[ot] OR underemployment[ti] OR underemployment[ot] OR Socioecono*[ti] OR Socioecono*[ot] OR "Socioeconomic Factors"[Mesh] OR "Work Engagement"[Mesh] OR career*[ti] OR career* [ot])))
- **QoL:** ("Quality of Life"[Mesh] OR Value of life[Ti] OR Value of life[ot] OR Health related quality of life[Ti] OR Health related quality of life[ot] OR HRQOL[Ti] OR HRQOL[ot] OR Quality of Life[Ti] OR Quality of Life[ot] OR QoL[Ti] OR QoL[ot] OR "Quality-Adjusted Life Years"[Mesh] OR QALY*[Ti] OR QALY*[ot] OR Quality adjusted life year*[Ti] OR Quality adjusted life year*[ot] OR Life quali*[Ti] OR Life quali*[ot])
- **Cognition:** (((Cognit*[Ti] OR Cognit*[ot] OR "Cognition"[Mesh] OR Memor*[Ti] OR Memor*[ot]) OR "Memory"[Mesh]))))
- **Autonomy:** ((("Dependency (Psychology)"[Mesh] OR Dependen* [ti] OR Dependen* [ot] OR indepen*[ti] OR indepen*[ot] OR Autonom*[Ti] OR Autonom*[ot] OR "Self Concept"[Mesh] OR "Personal Autonomy"[Mesh] OR Self esteem[ti] OR Self esteem[ot]))))
- **Participation:** ((participat*[Ti] OR participat*[ot] OR involve*[Ti] OR involve*[ot] OR "Social Participation"[Mesh] OR "Community Participation"[Mesh] OR "Stakeholder Participation"[Mesh]))))))

1B: Table with conducted searches in databases (Pubmed, Embase PsycInfo, Cochrane, Web of Science) and number of found studies per database.

Database	Studies	Search strategy
Pubmed	1421	(((((((((("Communication"[Mesh] OR Communicat*[Ti] OR Communicat*[ot]) OR conversat*[Ti] OR Conversat*[ot]))) OR ((("Work"[Mesh] OR "Employment"[Mesh] OR "Occupational Health" [Mesh] OR work*[Ti] OR work*[ot] OR labour*[Ti] OR labour*[ot] OR labor*[ti] OR labor*[ot] OR employment[ti] OR employment[ot] OR unemployment[ti] OR unemployment[ot] OR Occupation*[ti] OR Occupation*[ot] OR underemployment[ti] OR underemployment[ot] OR Socioecono*[ti] OR Socioecono*[ot] OR "Socioeconomic Factors"[Mesh] OR "Work Engagement"[Mesh] OR career*[ti] OR career* [ot]))))) OR (("Quality of Life"[Mesh] OR Value of life[Ti] OR Value of life[ot] OR Health related quality of life[Ti] OR Health related quality of life[ot] OR HRQOL[Ti] OR HRQOL[ot] OR Quality of Life[Ti] OR Quality of Life[ot] OR QoL[Ti] OR QoL[ot] OR "Quality-Adjusted Life Years"[Mesh] OR QALY*[Ti] OR QALY*[ot] OR Quality adjusted life year*[Ti] OR Quality adjusted life year*[ot] OR Life quali*[Ti] OR Life quali*[ot]))) OR ((((Cognit*[Ti] OR Cognit*[ot] OR "Cognition"[Mesh] OR Memor*[Ti] OR Memor*[ot]) OR "Memory"[Mesh])))))) OR (((("Dependency (Psychology)"[Mesh] OR Dependen* [ti] OR Dependen* [ot] OR indepen*[ti] OR indepen*[ot] OR Autonom*[Ti] OR Autonom*[ot] OR "Self Concept"[Mesh] OR "Personal Autonomy"[Mesh] OR Self esteem[ti] OR Self esteem[ot]))))))) OR (((participat*[Ti] OR participat*[ot] OR involve*[Ti] OR involve*[ot] OR "Social Participation"[Mesh] OR "Community Participation"[Mesh] OR "Stakeholder Participation"[Mesh]))))))))) AND (("Cochlear Implants"[Majr] OR Cochlear implantation[Majr] OR Cochlear implant*[ti] OR Cochlea implant*[ti] OR Cochlear Prosthe*[ti] OR Cochlea prosthe*[ti] OR Cochlear implant*[ot] OR Cochlea implant*[ot] OR Cochlear Prosthe*[ot] OR Cochlea prosthe*[ot]))) NOT (((("Child"[Mesh] OR "Infant"[Mesh] OR "Adolescent"[Mesh]) NOT ("Adult"[Mesh]))))
Embase	1432	((interpersonal communication/ or Communication barrier/ or communication skill/ or exp nonverbal communication/ or verbal communication/ or conversation/ or oral communication/ or verbal behavior/ or Communicat*.ti,kw. or conversat*.ti,kw. or exp "occupation and occupation related phenomena"/ or exp socioeconomics/ or exp social status/ or work*.ti,kw. or labour*.ti,kw. or labor*.ti,kw. or employment.ti,kw. or unemployment.ti,kw. or Occupation*.ti,kw. or underemployment.ti,kw. or Socioecono*.ti,kw. or Career*.ti,kw. or exp "quality of life"/ or Value of life.ti,kw. or Health related quality of life.ti,kw. or HRQOL.ti,kw. or Quality of Life.ti,kw. or QoL.ti,kw. or QALY*.ti,kw. or Quality adjusted life year*.ti,kw. or Life quali*.ti,kw. or cognition/ or exp memory/ or social cognition/ or Cognit*.ti,kw. or Memor*.ti,kw. or personal autonomy/ or exp self concept/ or dependent personality disorder/ or Dependen*.ti,kw. or indepen*.ti,kw. or Autonom*.ti,kw. or Self esteem.ti,kw. or social behavior/ or community participation/ or exp cooperation/ or exp group dynamics/ or job adaptation/ or exp social adaptation/ or social attitude/ or social interaction/ or social participation/ or stakeholder engagement/ or participat*.ti,kw. or involve*.ti,kw.) and (exp *cochlea prosthesis/ or *cochlear implantation/ or (Cochlea* implant* or Cochlea* Prosthe*).ti,kw.)) not (exp juvenile/ not exp adult/)
PsycInfo	1014	((exp communication/ or exp communication skills/ or communication barriers/ or Communicat*.ti,id. or conversat*.ti,ab,id. or exp occupations/ or career change/ or career development/ or exp job characteristics/ or occupational adjustment/ or occupational attitudes/ or occupational status/ or exp occupational stress/ or occupational success/ or exp socioeconomic status/ or Occupational Health/ or occupational mobility/ or career change/ or exp employment status/ or work*.ti,ab,id. or labour*.ti,ab,id. or labor*.ti,ab,id. or employment.ti,ab,id. or unemployment.ti,ab,id. or Occupation*.ti,ab,id. or underemployment.ti,ab,id. or Socioecono*.ti,ab,id. or Career*.ti,ab,id. or exp "quality of life"/ or exp life changes/ or well being/ or Value of life.ti,ab,id. or Health related quality of life.ti,ab,id. or HRQOL.ti,ab,id. or Quality of Life.ti,ab,id. or QoL.ti,ab,id. or QALY*.ti,ab,id. or Quality adjusted life year*.ti,ab,id. or Life quali*.ti,ab,id. or cognition/ or Cognitive Impairment/ or exp memory/ or social cognition/ or cognitive ability/ or verbal ability/ or Cognit*.ti,ab,id. or Memor*.ti,ab,id. or autonomy/ or "independence (personality)"/ or self-concept/ or self-confidence/ or self-esteem/ or "dependency (personality)"/ or Empowerment/ or Dependen*.ti,ab,id. or indepen*.ti,ab,id. or Autonom*.ti,ab,id. or Self esteem.ti,ab,id. or exp participation/ or exp involvement/ or participat*.ti,ab,id. or involve*.ti,ab,id.) and (cochlear implants/ or (Cochlea* implant* or Cochlea* Prosthe*).ti,ab,id.)) not ((("100" or "120" or "140" or "160" or "180" or "200") not ("300" or "320" or "340" or "360" or "380" or "390")).ag.)
Cochrane	184	(([mh "Communication"] OR Communicat*:ti,ab,kw OR conversat*:ti,ab,kw) OR ([mh "Work"] OR [mh "Employment"] OR [mh "Occupational Health"] OR work*:ti,ab,kw OR labour*:ti,ab,kw OR labor*:ti,ab,kw OR employment:ti,ab,kw OR unemployment:ti,ab,kw OR Occupation*:ti,ab,kw OR underemployment:ti,ab,kw OR Socioecono*:ti,ab,kw OR [mh "Socioeconomic Factors"] OR [mh "Work Engagement"] OR career*:ti,ab,kw) OR ([mh "Quality of Life"] OR Value of life:ti,ab,kw OR Health related quality of life:ti,ab,kw OR HRQOL:ti,ab,kw OR Quality of Life:ti,ab,kw OR QoL:ti,ab,kw OR [mh "Quality-Adjusted Life Years"] OR QALY*:ti,ab,kw OR Quality adjusted life year*:ti,ab,kw OR Life quali*:ti,ab,kw) OR (Cognit*:ti,ab,kw OR [mh "Cognition"] OR Memor*:ti,ab,kw OR [mh "Memory"]) OR ([mh "Dependency (Psychology)"] OR Dependen*:ti,ab,kw OR indepen*:ti,ab,kw OR Autonom*:ti,ab,kw OR [mh "Self Concept"] OR [mh "Personal Autonomy"] OR Self esteem:ti,ab,kw) OR (participat*:ti,ab,kw OR involve*:ti,ab,kw OR [mh "Social Participation"] OR [mh "Community Participation"] OR [mh "Stakeholder Participation"])) AND ([mh "Cochlear Implants"] OR [mh "Cochlear implantation"] OR Cochlear implant*:ti,ab,kw OR Cochlea implant*:ti,ab,kw OR Cochlear Prosthe*:ti,ab,kw OR Cochlea prosthe*:ti,ab,kw) NOT ([mh child] OR [mh infant] OR [mh adolescent]) NOT ([mh Adult])
Web of Science	1,916	((TS=(Communicat*) OR TS=(conversat*) OR TS= (work*) OR TS= (labour*) OR TS=(labor*) OR TS= (employment) OR TS= (unemployment) OR TS=(Occupation*) OR TS=(underemployment) OR TS=(Socioecono*) OR TS=(career*) OR TS=("Value of life") OR TS=("Health related quality of life") OR TS=(HRQOL) OR TS=("Quality of Life") OR TS=(QoL) OR TS=(QALY*) OR TS=("Quality adjusted life year*") OR TS=("Life quali*") OR TS=(Cognit*) OR TS=(Memor*) OR TS=(Dependen*) OR TS=(indepen*) OR TS=(Autonom*) OR TS=("Self esteem") OR TS=(participat*) OR TS=(involve*)) AND (TI= (Cochlear implant*) OR TI= (Cochlea implant*) OR TI= (Cochlear Prosthe*) OR TI= (Cochlea Prosthe*)) NOT ((TS=(Child*) OR TS=(juveni*) OR TS=(Infan*) OR TS=(adolescent)) NOT (TS=(Adult*) OR TS=(elder*) OR TS=(matur*) OR TS=(grown*) OR TS=(aged*)))) OR ((TI=(Communicat*) OR TI=(conversat*) OR TI= (work*) OR TI= (labour*) OR TI=(labor*) OR TI= (employment) OR TI= (unemployment) OR TI=(Occupation*) OR TI=(underemployment) OR TI=(Socioecono*) OR TI=(career*) OR TI=("Value of life") OR TI=("Health related quality of life") OR TI=(HRQOL) OR TI=("Quality of Life") OR TI=(QoL) OR TI=(QALY*) OR TI=("Quality adjusted life year*") OR TI=("Life quali*") OR TI=(Cognit*) OR TI=(Memor*) OR TI=(Dependen*) OR TI=(indepen*) OR TI=(Autonom*) OR TI=("Self esteem") OR TI=(participat*) OR TI=(involve*)) AND (TI= (Cochlear implant*) OR TI= (Cochlea implant*) OR TI= (Cochlear Prosthe*) OR TI= (Cochlea Prosthe*)))

1C: Updated search strategy on 15-4-2020 pubmed; this strategy was translated to the other four databases.

Patient: NOT (((("Child"[Mesh] OR "Infant"[Mesh] OR "Adolescent"[Mesh]) NOT ("Adult"[Mesh])))))
Intervention: (("Cochlear Implants"[Majr] OR Cochlear implantation[Majr] OR Cochlear implant*[ti] OR Cochlea implant*[ti] OR Cochlear Prosthe*[ti] OR Cochlea prosthe*[ti] OR Cochlear implant*[ot] OR Cochlea implant*[ot] OR Cochlear Prosthe*[ot] OR Cochlea prosthe*[ot])))
Comparison: N/A
Outcomes:

- **Work**: ((("Work"[Mesh] OR "Employment"[Mesh] OR "Occupational Health" [Mesh] OR work*[Ti] OR work*[ot] OR labour*[Ti] OR labour*[ot] OR labor*[ti] OR labor*[ot] OR employment[ti] OR employment[ot] OR unemployment[ti] OR unemployment[ot] OR Occupation*[ti] OR Occupation*[ot] OR underemployment[ti] OR underemployment[ot] OR Socioecono*[ti] OR Socioecono*[ot] OR "Socioeconomic Factors"[Mesh] OR "Work Engagement"[Mesh] OR career*[ti] OR career* [ot])))))
- **Autonomy:** (((("Dependency (Psychology)"[Mesh] OR Dependen* [ti] OR Dependen* [ot] OR indepen*[ti] OR indepen*[ot] OR Autonom*[Ti] OR Autonom*[ot] OR "Self Concept"[Mesh] OR "Personal Autonomy"[Mesh] OR Self esteem[ti] OR Self esteem[ot]))))))
- **Participation:** (((participat*[Ti] OR participat*[ot] OR involve*[Ti] OR involve*[ot] OR "Social Participation"[Mesh] OR "Community Participation"[Mesh] OR "Stakeholder Participation"[Mesh])))))))))

Filters: **Publication date from 2019/06/04 to 2020/04/31**

1D:Table with conducted searches on 15-4-2020 in the five databases and number of found studies per database between June 2019 and April 2020.

Database	Studies	Search strategy
Pubmed	15	(((((((((("Work"[Mesh] OR "Employment"[Mesh] OR "Occupational Health" [Mesh] OR work*[Ti] OR work*[ot] OR labour*[Ti] OR labour*[ot] OR labor*[ti] OR labor*[ot] OR employment[ti] OR employment[ot] OR unemployment[ti] OR unemployment[ot] OR Occupation*[ti] OR Occupation*[ot] OR underemployment[ti] OR underemployment[ot] OR Socioecono*[ti] OR Socioecono*[ot] OR "Socioeconomic Factors"[Mesh] OR "Work Engagement"[Mesh] OR career*[ti] OR career* [ot]))))) OR (((("Dependency (Psychology)"[Mesh] OR Dependen* [ti] OR Dependen* [ot] OR indepen*[ti] OR indepen*[ot] OR Autonom*[Ti] OR Autonom*[ot] OR "Self Concept"[Mesh] OR "Personal Autonomy"[Mesh] OR Self esteem[ti] OR Self esteem[ot])))))) OR (((participat*[Ti] OR participat*[ot] OR involve*[Ti] OR involve*[ot] OR "Social Participation"[Mesh] OR "Community Participation"[Mesh] OR "Stakeholder Participation"[Mesh]))))))))) AND (("Cochlear Implants"[Majr] OR Cochlear implantation[Majr] OR Cochlear implant*[ti] OR Cochlea implant*[ti] OR Cochlear Prosthe*[ti] OR Cochlea prosthe*[ti] OR Cochlear implant*[ot] OR Cochlea implant*[ot] OR Cochlear Prosthe*[ot] OR Cochlea prosthe*[ot]))) NOT (((("Child"[Mesh] OR "Infant"[Mesh] OR "Adolescent"[Mesh]) NOT ("Adult"[Mesh]))))) Filters: Publication date from 2019/06/04 to 2020/04/31
Embase	45	((exp "occupation and occupation related phenomena"/ or exp socioeconomics/ or exp social status/ or work*.ti,kw. or labour*.ti,kw. or labor*.ti,kw. or employment.ti,kw. or unemployment.ti,kw. or Occupation*.ti,kw. or underemployment.ti,kw. or Socioecono*.ti,kw. or Career*.ti,kw. or personal autonomy/ or exp self concept/ or dependent personality disorder/ or Dependen*.ti,kw. or indepen*.ti,kw. or Autonom*.ti,kw. or Self esteem.ti,kw. or social behavior/ or community participation/ or exp cooperation/ or exp group dynamics/ or job adaptation/ or exp social adaptation/ or social attitude/ or social interaction/ or social participation/ or stakeholder engagement/ or participat*.ti,kw. or involve*.ti,kw.) and (exp *cochlea prosthesis/ or *cochlear implantation/ or (Cochlea* implant* or Cochlea* Prosthe*).ti,kw.)) not (exp juvenile/ not exp adult/)<br>limit 1 to yr="2019 -Current"
PsycInfo	37	((exp occupations/ or career change/ or career development/ or exp job characteristics/ or occupational adjustment/ or occupational attitudes/ or occupational status/ or exp occupational stress/ or occupational success/ or exp socioeconomic status/ or Occupational Health/ or occupational mobility/ or career change/ or exp employment status/ or work*.ti,ab,id. or labour*.ti,ab,id. or labor*.ti,ab,id. or employment.ti,ab,id. or unemployment.ti,ab,id. or Occupation*.ti,ab,id. or underemployment.ti,ab,id. or Socioecono*.ti,ab,id. or Career*.ti,ab,id. or autonomy/ or "independence (personality)"/ or self-concept/ or self-confidence/ or self-esteem/ or "dependency (personality)"/ or Empowerment/ or Dependen*.ti,ab,id. or indepen*.ti,ab,id. or Autonom*.ti,ab,id. or Self esteem.ti,ab,id. or exp participation/ or exp involvement/ or participat*.ti,ab,id. or involve*.ti,ab,id.) and (cochlear implants/ or (Cochlea* implant* or Cochlea* Prosthe*).ti,ab,id.)) not (("100" or "120" or "140" or "160" or "180" or "200") not ("300" or "320" or "340" or "360" or "380" or "390")).ag.<br>limit 3 to yr="2019 -Current"
Cochrane	16	((([mh "Work"] OR [mh "Employment"] OR [mh "Occupational Health"] OR work*:ti,ab,kw OR labour*:ti,ab,kw OR labor*:ti,ab,kw OR employment:ti,ab,kw OR unemployment:ti,ab,kw OR Occupation*:ti,ab,kw OR underemployment:ti,ab,kw OR Socioecono*:ti,ab,kw OR [mh "Socioeconomic Factors"] OR [mh "Work Engagement"] OR career*:ti,ab,kw) OR ([mh "Dependency (Psychology)"] OR Dependen*:ti,ab,kw OR indepen*:ti,ab,kw OR Autonom*:ti,ab,kw OR [mh "Self Concept"] OR [mh "Personal Autonomy"] OR Self esteem:ti,ab,kw) OR (participat*:ti,ab,kw OR involve*:ti,ab,kw OR [mh "Social Participation"] OR [mh "Community Participation"] OR [mh "Stakeholder Participation"])) AND ([mh "Cochlear Implants"] OR [mh "Cochlear implantation"] OR Cochlear implant*:ti,ab,kw OR Cochlea implant*:ti,ab,kw OR Cochlear Prosthe*:ti,ab,kw OR Cochlea prosthe*:ti,ab,kw) NOT ([mh child] OR [mh infant] OR [mh adolescent]) NOT ([mh Adult]))<br>with Cochrane Library publication date from Jun 2019 to May 2020
Web of Science	155	((TS= (work*) OR TS= (labour*) OR TS=(labor*) OR TS= (employment) OR TS= (unemployment) OR TS=(Occupation*) OR TS=(underemployment) OR TS=(Socioecono*) OR TS=(career*) OR TS=(Dependen*) OR TS=(indepen*) OR TS=(Autonom*) OR TS=("Self esteem") OR TS=(participat*) OR TS=(involve*)) AND (TI= (Cochlear implant*) OR TI= (Cochlea implant*) OR TI= (Cochlear Prosthe*) OR TI= (Cochlea Prosthe*)) NOT ((TS=(Child*) OR TS=(juveni*) OR TS=(Infan*) OR TS=(adolescent)) NOT (TS=(Adult*) OR TS=(elder*) OR TS=(matur*) OR TS=(grown*) OR TS=(aged*)))) OR ((TI= (work*) OR TI= (labour*) OR TI=(labor*) OR TI= (employment) OR TI= (unemployment) OR TI=(Occupation*) OR TI=(underemployment) OR TI=(Socioecono*) OR TI=(career*) OR TI=(Dependen*) OR TI=(indepen*) OR TI=(Autonom*) OR TI=("Self esteem") OR TI=(participat*) OR TI=(involve*)) AND (TI= (Cochlear implant*) OR TI= (Cochlea implant*) OR TI= (Cochlear Prosthe*) OR TI= (Cochlea Prosthe*)))<br>Indexes=SCI-EXPANDED, SSCI, A&HCI, CPCI-S, CPCI-SSH, BKCI-S, BKCI-SSH, ESCI, CCR-EXPANDED, IC Timespan=2019-2020
